# Nanotechnology for the treatment of coronary in stent restenosis: a clinical perspective

**DOI:** 10.1186/2045-824X-3-8

**Published:** 2011-04-18

**Authors:** Garry McDowell, Mark Slevin, Jerzy Krupinski

**Affiliations:** 1 Faculty of Health, Edge Hill University, Ormskirk, UK; 2 School of Biology, Chemistry and Health Science, John Dalton Building, Manchester Metropolitan University, Chester Street, Manchester, M1 5GD; 3 Cardiovascular Research Centre, CSIC-ICCC, Hospital de la Santa Creu i Sant Pau, Barcelona, Spain; 4 Division of Cerebrovascular Diseases, Department of Neurology, Hospital Universitari Mútua de Terrassa, Barcelona, Spain

## Abstract

Coronary in stent restenosis remains a significant limitation to the long term efficacy of coronary artery stent placement. In this review the authors review the pathophysiology of coronary in stent restenosis, together with an overview of the current treatment modalities. The potential clinical utility of nanotechnology is also reviewed.

The first human safety trial of systemic nanoparticle paclitaxel (nab-paclitaxel) for in stent restenosis (SNAPIST-I) is discussed. The results showed no significant adverse advents attributable to the nab-paclitaxel at 10 or 30 mg/m^2^, although moderate neutropenia, sensory neuropathy and mild to moderate reversible alopecia occurred at higher doses. No major adverse cardiac events were recorded at 2 months, whilst at 6 months, 4 target lesions required revascularisation. The investigators concluded therefore that systemic nab-paclitaxel was well tolerated at a dose of <70 mg/m^2^. To date however, no formal clinical evaluation has been reported as to the clinical utility of nab-paclitaxel, or any of the nano preparations discussed, for the suppression of coronary in stent restenosis.

## 1. Introduction: Overview of Nanomedicine Applications in Cardiology

Cardiovascular disease, including acute coronary syndromes and cerebrovascular events continue to be a major source of mortality and morbidity. Current medical screening and diagnosis is limited and many of the symptoms and signs of cardiovascular and cerebrovascular disease are non-specific.

Nanomedicine provides a unique opportunity to explore at a cellular or organ level the various pathophysiologies of the cardiovascular system. Nanomolecules have been used in:

• Assessing and treating atherosclerosis in asymptomatic patients

• Coronary revasculariation

• Thrombolytic therapy

• Treatment of coronary in stent restenosis.

## 2. The use of Nanotechnology for the Treatment of Coronary In Stent Restenosis

There is still a significant requirement for a novel drug delivery mechanism for the treatment of coronary in stent restenosis, due to the limitations of the current modalities including late stent thrombosis.

A nanoparticle based approach is ideal for the treatment of restenosis since targeted delivery of nanoparticles is feasible and much lower concentrations of the active drug can be used hence reducing systemic toxicity.

The size of particle however is critical in the distribution of nanoparticles in the blood vessel wall. Westedt *et al *[1], in experiments conducted using the aorta abdominalis of New Zealand White Rabbits as a model system, report that nanoparticles of 100 and 200 nm are able to penetrate to the inner layers of the vessel wall, while 514 nm particles accumulate predominantly at the luminal surface.

Some examples of nanoparticles used in the treatment of in stent restenosis are reviewed below.

### 3.1 Lipid Based Nanoparticles

Liposomes are small and have a spherical shape and are formed from natural and non-toxic phospholipids and cholesterol. As liposomes are small and posses hydrophobic and hydrophilic characteristics there are ideally suited to the development of novel drug delivery systems [2]. Liposome surfaces can be modified to increase circulating half-life and conjugated to antibodies or ligands for enhanced tissue specificity.

Lipid based nanoparticles have been utilised to deliver a number of different classes of drug to the arterial endothelium.

Clodronate, a bisphonsphonate, has been delivered using liposome nanoparticle of 1:3 distearoyl phosphatidylglycerol, 1, 2 distearoly-sn-gylcero-3-phosphcholine [3]. Lyposomal choldronate inhibited neointimal growth in the balloon injured rabbit carotid artery after systemic administration. Other members of the bisphosphonate class of drugs including pamidronate and alendronate have been utilised as antirestenotic agents in balloon injured rat carotid artery model [4]. It is noteworthy, however, that these experiments were performed in a carotid artery model and whether the results are relevant to coronary restenosis after PCI remains unknown.

TRM 484 consists of nanoparticles of prednisolone with high infinity to chrondroitin suphate proteoglycans and at a dose of 1 mg/kg significantly reduced neo intimal growth in atherosclaratic New Zealand White Rabbits implanted with bare metal stents [5].

### 3.2 Polymeric based nanoparticles

These are solid, colloidal particles of macromolecules that range in size from 10-1000 nm [6]. They are idea drug delivery systems [7], where the compound of interest may be dissolved, entrapped, adsorbed, attached or encapsulated into the nanoparticle matrix [8,9].

Early work on polymeric nanoparticles began with a comparison of probucol delivery by polymeric and liposomal nanoparticles. Probucol has been shown to reduce restenosis after angioplasty provided oral administration is commenced one month before the procedure [10]. Klughertz and colleagues prepared ^35^S-probucol incapsulated in either liposomal or polylactic-coglycolic acid (PLGA) nanoparticles, which were delivered using an infusion catheter after balloon angioplasty of rabbit iliac arteries. Iliac arteries, perivascular fat and downstream tissues were harvested and the radioactivity measured from animals euthanized on day 0,3 and 7 after dosing. The results showed after delivery efficiency was superior with PLGA [10]. It should be noted however that these experiments were conducted outside the coronary circulation.

Cohen Sela *et al *reported that PLGA nanoparticles containing alendronate reduced neointimal formation and restenosis by systemic transient depletion of monocytes in hypercholesterolaemic rabbit model [11]. Further work by the same group reported incorporation of the bisphosphonates, 2-(2-Aminopyrimidino) ethyldiene-1, 1-bisphosphonbic acid betaine (ISA) into PLGA based nanoparticles (ISA - NP) [12]. Intravenously administered ISA-NP resulted in a significant attenuation of restenosis by 45%, 14-days after carotid artery injury in comparison to a control group of animals treated with free ISA, buffer or blank nanoparticles. However, the effect was not preserved long-term (30-days post injury) and no significant reduction in neointimal reduction was observed. Surprisingly, significant neointimal suppression was observed following subcutaneous injection of ISA-NP [12].

Paclitaxel is a member of the taxane family of drugs. Paclitaxel loaded nanoparticles have been prepared from oil-water emulsion using biodegradable PLGA and surface modified with the cationic surfactant didoceylmethylammonium bromide (DMAB) to enhance arterial retention. *In vivo *investigations have been performed in balloon injured rabbit carotid arteries treated with a single infusion of paclitaxel loaded nanoparticles and observed for 28 days. The results demonstrated that the inhibitory effect on intimal proliferation was dose dependant, and at 30 mg/ml nanoparticle concentration, completely inhibited intimal proliferation, leading the group to speculate that the surface modified paclitaxel loaded nanoparticles provide an effective means of inhibiting the proliferative response to vascular injury [13]. Further results by Westedt *et al *[14] substantiated these findings utilising paclitaxel-loaded nanoparticles administered locally to the wall of balloon injured rabbit iliac arteries using a porous balloon catheter. The results demonstrated a 50% reduction in neointimal area compared to the control vessels treated with blank nanoparticles.

Work from another group has demonstrated that the antiproliferative effects of paclitaxel can be significantly improved by co-administration of other agents [15]. C6-ceramide is an apoptotic signalling molecule and has been combined with paclitaxel in polymeric nanoparticles consisting of poly(ethylene oxide) - modified poly (episilon caprolactone). Combination of paclitaxel with cereamide when administered in nanoparticle formulation significantly augmented the antiproliferative effect of either agent alone [15].

The angiotensin-converting-enzyme inhibitor, lisinopril, has also been encapsulated in nanoparticles of PLGA for site specific delivery by catheters for the prevention of coronary in stent restenosis [16], although to date *in-vivo *studies to examine the anti-restenotic effect have not been reported.

Further work from Cohen-Sela's group [17] incorporated the antiproliferative agent mitramycin into PLGA nanoparticles using a nanoparticipation technique. Unfortunately *in-vivo *testing using a rat carotid artery model showed no inhibition of restenosis. The authors suggest that this is probably due to the short depletion period of circulating monocytes and the lack of arterial targeting.

Work to increase the bioadhesive properties of nanoparticles has been suggested to improve retention and arterial uptake of nanoparticles into the arterial wall [18]. Zou *et al *[18] prepared bioadhesive PLGA nanoparticles, encapsulating rapamycin, using different concentration of carbopol 940, however *in-vivo *results are awaited.

Recent research has focused on the administration of drugs using biodegradable polymer nanoparticles capable of prolonged drug release. Sustained drug release of dexamethasone or rapamycin from nanoparticles based on poly (ethylene oxide) and poly (d.L-lactic-co-glycolic acid) block copolymers has been investigated [19]. The investigators found that treating the nanoparticles with gelatine or albumin after drug loading resulted in a linear drug release, the rate of release being related to the amount of protein associated with the nanoparticles [19]. Release of dexamethasone and rapamycin was sustained for 17 and 50 days respectively [19].

Luderer *et al *[20] report the use of sirolimus loaded biodegradable poly (D,L lactide) nanoparticles as drug carriers to prevent restenosis following coronary angioplasty [20]. The particles showed a biphasic release pattern consisting of a short burst release of 50% w/w sirolimus followed by a longer slower release.

Moreover, Nakano *et al *[21] have succeeded in formulating a nanoparticle eluting stent. In a porcine coronary artery model, the magnitude of stent induced injury, inflammation, endothelial recovery and neointimal formation were comparable between bare metal stent and nanoparticle eluting stent. It is worthy of note, however, that the study neither presents data on restenosis rate nor the incorporation of any pharmaceutical preparations within the nanoparticles.

The tryphostins are a class of platelet derived growth factor (PDGF) receptor β tyrosine kinase blockers [22,23]. Preclinical investigations have reported results with the experimental compound AG-1295 incorporated in polylactide nanoparticles. PLA AG-1295 nanoparticles were delivered via an infusion catheter in a balloon injured swine model, resulting in inhibition of smooth muscle cell (SMC) growth. Further, another tyrphostin AGL-2043 encapsulated in in PLA nanoparticles inhibited restenosis in both balloon injured rat carotid artery and stented porcine artery models [24].

### 3.3 Gel like Nanoparticles

Previous research [25] has demonstrated that nanosized (100 nm) hydrogel spheres made of poly (N-isopropylacrylamide) are internalised by endothelial cells and VSMC more than microspheres (1 μm), although cellular uptake was dependant on the incubation time, sphere concentration and introduced shear stress levels of the medium. In contrast, microspheres were rapidly taken up by phagocytes, especially at high concentration [25]. These findings lead the authors to suggest that hydrogel nanospheres are more effective as an intravascular delivery system in terms of vascular uptake and biocompatibility [25].

Since significant number of VSMC undergo rapid apoptosis following balloon angioplasty Reddy and colleagues [26] tested the hypothesis that preventing VSMC from apoptosis could prevent intimal hyperplasia. They used rapamycin (which has anti-apoptotic and antiproliferative actions) loaded gel nanoparticles of mean diameter 54 nm. When infused into a rat carotid artery model of vascular injury the authors report significant inhibition of hyperplasia and re-endothelialisation of the injured artery. Further, the group report inhibition of activation of caspase 3/7 enzyme systems in the treated artery, preventing VSMC from undergoing apoptosis [26]

### 3.4 Miscellaneous Studies

Kolodgie *et al *[27] report the preparation of paclitaxel loaded albumin based nanoparticles for the reduction of in stent neointimal growth. The research conducted in New Zealand White Rabbits receiving bilateral iliac artery stents yielded significant results. Systemic administration of albumin nanoparticles containing paclitaxel reduced neointimal growth at 28 days. A further single repeated dose was required for sustained neointimal suppression at 90 days post procedure [27].

Moreover, further preclinical work has demonstrated the utility of tissue factor targeted nanoparticles containing doxorubicin or paclitaxel to inhibit VSMC proliferation in culture [28].

In addition, intra mural delivery of αVβ3-targeted rapamycin loaded nanoparticles inhibited stenosis without delaying endothelial healing after balloon injury [29].

Chorney *et al *[30] report the use of uniform magnetic fields to control the release of paclitaxel from biocompatible magnetic nanoparticles. The research showed that magnetic treatment of cultured arterial SMC with paclitaxel loaded magnetic nanoparticles caused significant inhibition of cell growth, which was not observed under non-magnetic conditions. The authors postulate that the results demonstrate the feasibility of site specific drug delivery by uniform field controlled targeting of magnetic nanopatticles [30].

### 3.5 Clinical Study

To date only one human study has been reported in the literature. In 2007, Margolis *et al *[31] presented the first human safety trial of systemic nanoparticle paclitaxel (nab-paclitaxel) for in stent restenosis I (SNAPIST-I). In this study the investigators administered systemic treatment with a 130 nm albumin nanoparticle encapsulating paclitaxel in 10, 30, 70 and 100 mg/m^2 ^intravenously after stenting of a single lesions of ≥ 3 mm in 23 patients. The results showed no significant adverse events attributable to the nab-paclitaxel at 10 or 30 mg/m^2^, although moderate neutropenia, sensory neuropathy and mild to moderate reversible alopecia occurred at higher doses. No major adverse cardiac events were recorded at 2 months, whilst at 6 months 4 target lesions required revascularisation. The investigators concluded therefore that systemic nab-paclitaxel was well tolerated at a dose of <70 mg/m^2 ^[31]. To date however no formal clinical evaluation has been reported as to the clinical utility of nab-paclitaxel for the suppression of coronary in stent restenosis.

## 4. Conclusion

Much work therefore has been undertaken to evaluate the potential clinical utility of nanoparticles for the targeted and non-targeted delivery of various agents with antiproliferative and anti-restenostic actions. To date most of these investigations have been conducted either *in-vitro *or *in-vivo *utilising animal models. Many studies have also been conducted outside the coronary circulation and hence the relevance of the result to coronary in stent restenosis can only be postulated. To date only one human study has been reported. This was a dose ranging study demonstrating that systemic nab-paclitaxel was well tolerated at a dose of <70 mg/m^2 ^[23]. No formal clinical evaluation has been reported as to the clinical utility of nab-paclitaxel for the suppression of coronary in stent restenosis.

## Competing interests

The authors declare that they have no competing interests.

## Authors' contributions

GMcD: Contributed to the background research, writing and critical review of the manuscript. Read and approved the final manuscript

MS: Contributed to the background research, writing and critical review of the manuscript. Read and approved the final manuscript.

JS: Contributed to the critical review of the manuscript. Read and approved the final manuscript.

All authors read and approved the final manuscript.
